# Development and Application of a Life Review Program to Support Mental Health Among Middle-Aged and Older Adults Living with HIV in Taiwan

**DOI:** 10.3390/healthcare13141698

**Published:** 2025-07-15

**Authors:** Hong Hong

**Affiliations:** Bachelor Program of Senior Health Promotion and Care Management for Indigenous People, National Changhua University of Education, Changhua 50007, Taiwan; oscarhong@cc.ncue.edu.tw

**Keywords:** Swanson’s theory of caring, narrative-based intervention, psychosocial support, life review program, HIV and aging

## Abstract

**Background**: This article introduces the development and application of a life review program designed to support the psychological well-being of middle-aged and older adults living with HIV. Grounded in Swanson’s Theory of Caring, the program emphasizes culturally sensitive, narrative-based reflection and group sharing. **Methods**: This study employed a qualitative exploratory design, implementing an eight-week life review program based on Swanson’s Theory of Caring. Data were collected through in-depth interviews and participant observations and then analyzed using qualitative content analysis. **Results**: Thematic analysis revealed that participants experienced emotional engagement, renewed self-understanding, and a sense of connection fostered by the caring-based narrative process. Participants also expressed positive attitudes toward reinterpreting their past and envisioning the future. **Conclusions**: Rather than evaluating intervention outcomes, this study focuses on the development and implementation of a caring-based life review tool. Findings illustrate its feasibility and cultural acceptability, offering a foundation for future adaptation across diverse supportive care settings. This culturally sensitive intervention represents one of the first applications of a caring-based life review program tailored to aging PLWHIV in Taiwan.

## 1. Introduction

People living with HIV (PLWHIV) face not only medical challenges but also profound psychological and social difficulties. The physiological impact of the virus and the side effects of long-term antiretroviral therapy can contribute to chronic health issues, while stigma, discrimination, and social isolation further exacerbate psychological distress [1,2]. Studies indicate that PLWHIV experience significantly higher rates of depression and anxiety compared to the general population, affecting adherence to antiretroviral therapy and overall health outcomes [3,4].

In Taiwan, although significant progress has been made toward achieving the UNAIDS 90-90-90 targets, mental health services for PLWHIV remain underdeveloped. Reports from the Taiwan Centers for Disease Control [5] highlight that psychological support is a critical unmet need among middle-aged and older PLWHIV, who often experience compounded trauma from life events such as drug addiction, incarceration, hospitalization, and familial rejection.

Therefore, addressing the psychological well-being of PLWHIV is not merely an adjunct to medical treatment but an essential component of holistic HIV care. Given the growing number of middle-aged and older PLWHIV worldwide, there is an urgent need for interventions that are developmentally appropriate, culturally sensitive, and resilience-focused [6,7].

Various interventions have been developed to address the mental health needs of PLWHIV, including cognitive behavioral therapy (CBT), support groups, peer counseling, and digital health applications. Cognitive behavioral interventions have shown effectiveness in reducing depressive symptoms and improving medication adherence among PLWHIV [8,9]. Peer support models, such as the “Positive Peers” intervention, have been associated with enhanced social connectedness and reduced internalized stigma [10]. Additionally, digital interventions, including telemedicine and mobile health (mHealth) programs, have expanded access to psychological services for PLWHIV, especially in resource-limited settings [11,12].

Despite these advancements, most interventions have primarily focused on general adult populations, with limited attention to the unique needs of middle-aged and older PLWHIV. Research indicates that aging PLWHIV face distinct psychosocial challenges, including compounded stigma related to both HIV status and aging, cumulative health burdens, and greater risks of isolation and depression [13,14,15]. Furthermore, existing interventions often overlook culturally sensitive and narrative-based approaches that can facilitate emotional expression, strengthen resilience, and foster self-acceptance—critical elements for long-term psychological well-being in this population.

In Taiwan, integrated psychological services tailored to aging PLWHIV remain scarce. A recent study on mental health services for people living with HIV in Taiwan highlighted a lack of culturally adapted, life-course-sensitive interventions [16]. These gaps underscore the urgent need for innovative, culturally responsive programs that not only address clinical symptoms but also empower middle-aged and older PLWHIV to reconstruct meaning and enhance life satisfaction.

Narrative-based interventions, such as life review therapy, have been recognized as effective strategies for promoting psychological well-being in populations facing chronic illnesses and aging-related challenges. By facilitating the reconstruction of personal meaning and fostering emotional expression, life review interventions can help individuals integrate past experiences, enhance resilience, and restore a sense of self-worth [17,18]. For middle-aged and older PLWHIV, who often contend with cumulative trauma, societal stigma, and social isolation, life review provides an opportunity to reinterpret their life stories positively and to reclaim agency in the face of adversity [19].

Research has demonstrated that life review interventions are associated with reductions in depressive symptoms, improvements in quality of life, and enhanced coping capacities among older adults [20,21]. Despite its demonstrated effectiveness in aging populations, the application of narrative-based approaches among aging PLWHIV remains scarce, particularly within culturally specific contexts such as Taiwan.

This study aims to introduce the development and application of a culturally sensitive life review program designed to support the mental health of middle-aged and older PLWHIV in Taiwan. By describing the program structure and summarizing the participants’ experiential feedback, this study seeks to contribute to the growing field of narrative-based interventions for aging and marginalized populations.

In light of these challenges, there is a pressing need for psychosocial support tools that are both theory-driven and culturally grounded [22,23]. This study presents the development and application of a life review program designed to support the psychological well-being of middle-aged and older adults living with HIV in Taiwan. The program was developed based on Swanson’s Theory of Caring [24], integrating narrative-based reflection and culturally responsive elements to foster emotional processing, self-worth, and social connection. Rather than evaluating intervention efficacy, this study focuses on introducing the structure of the program and illustrating its application through a small pilot implementation with six participants. The purpose is to demonstrate the feasibility and potential relevance of this caring-based tool for long-term supportive care in marginalized aging populations.

## 2. Methods

### 2.1. Program Development

The life review program was conceptually developed based on Swanson’s Theory of Caring, which identifies five key caring processes: knowing, being with, doing for, enabling, and maintaining belief. These processes were used to guide the structure, content, and facilitation strategies of the program to ensure that emotional and relational dimensions of care were embedded throughout.

Each of the eight weekly sessions was designed to align with one or more caring processes. For example, “knowing” was operationalized through early sessions involving the exploration of personal histories and life turning points; “being with” was expressed in shared group dialogues that provided space for empathic presence; “doing for” appeared in creative activities such as life mapping and guided storytelling; “enabling” was fostered through strengths-based reflection and future goal setting; and “maintaining belief” was supported by affirming participants’ dignity, hope, and inner resilience, especially in the face of cumulative trauma.

Cultural sensitivity was also a core design principle. The program incorporated local language references, respect for collectivist cultural values, and age-appropriate metaphors relevant to Taiwanese PLWHIV. Session themes included self-introduction, personal strengths, life struggles, emotional expression, and envisioning the future. Sensory elements such as music, essential oils, photographs, and symbolic objects were integrated to promote memory recall and emotional engagement. Notably, most participants (83.3%) reported HIV transmission through intravenous drug use, reflecting a common transmission route among aging PLWHIV in Taiwan (Table 1).

### 2.2. Research Design Justification

This study employed a qualitative, exploratory design aimed at understanding the subjective psychological experiences of middle-aged and older adults living with HIV following their participation in a life review intervention. As the primary objective was to explore emotional meaning-making, resilience, and perceived transformation, the study did not include standardized pre- and post-intervention psychological assessments. Instead, rich narrative data were collected through in-depth interviews and participant observation. This design is particularly appropriate for pilot-stage research in underexplored populations, such as aging PLWHIV in Taiwan, where limited evidence exists regarding culturally grounded narrative-based interventions.

### 2.3. Participants and Setting

Participants were recruited from two long-term care facilities in central Taiwan that provided specialized services for people living with HIV. Inclusion criteria were: (1) aged 45 years or older, (2) confirmed diagnosis of HIV, (3) ability to provide informed consent, and (4) willingness to participate in weekly sessions. A total of six participants were enrolled. Participants were referred by facility directors and social workers, who identified individuals perceived as emotionally distressed, socially withdrawn, or psychologically vulnerable based on their professional judgment and long-term interactions. Although no standardized psychological assessments were conducted prior to participation, purposive sampling focused on individuals who were likely to benefit from a reflective and supportive intervention. Table 2 presents the detailed demographic characteristics of each participant.

### 2.4. Ethical Considerations

This study adheres to the principles of the Declaration of Helsinki and has received approval from the National Cheng Kung University Ethics Review Committee (Approval Number: NCKU-IRB-113-101).

### 2.5. Data Collection

Data were collected through semi-structured interviews conducted at baseline and after the completion of the program, as well as through participant observations during the sessions. Interviews explored participants’ emotional experiences, perceived changes, and reflections on the intervention. All interviews were audio-recorded with consent and transcribed verbatim for analysis. Field notes were also maintained to capture non-verbal cues and group dynamics during sessions.

### 2.6. Data Analysis

A qualitative content analysis approach was employed, following Graneheim and Lundman’s (2004) method, which involves identifying meaning units, condensing them into codes, and categorizing them into themes [25]. This process ensured a systematic and rigorous analysis of participant narratives and observational data. Thematic analysis was conducted to identify recurring patterns, themes, and emotional responses. Coding was performed independently by two researchers to enhance reliability, and discrepancies were resolved through discussion. Key themes were organized to reflect the core emotional and psychological changes experienced by participants during the program.

### 2.7. Trustworthiness and Rigor

We evaluated the rigor and trustworthiness of this study based on the four criteria for qualitative research accuracy proposed by Guba and Lincoln [26]: (1) Credibility: The researchers have extensive experience in studying PLWH’s mental health and have received certification from the Taiwan Ministry of Health and Welfare on HIV prevention knowledge and protective measures (Long-Term Care Professionals Digital Learning, Certificate No. 20220803018). Additionally, the researchers underwent comprehensive training in qualitative research and possessed expertise in interviews and qualitative analysis. Regular discussions with qualitative research experts were also an essential part of the research process. (2) Transferability: The interview content was accurately and faithfully transcribed verbatim to ensure it could be properly presented in this study. The transcripts were returned to the participants for verification and correction. (3) Dependability: We invited two professionals with extensive experience in qualitative research and HIV studies to review and revise the categorization of the research findings. (4) Confirmability: The researchers maintained all reflective field notes and data analysis records for future validation and reference. In the final stage of the study, participants were given the opportunity to review and confirm the research findings.

## 3. Results

To provide context for the participants’ experiences described below, a brief summary of the life review program is presented. The intervention was structured as an eight-week group-based program, designed according to Swanson’s Theory of Caring, which emphasizes five core processes: knowing, being with, doing for, enabling, and maintaining belief. Each session incorporated narrative reflection, culturally adapted activities, and sensory stimuli—such as music, essential oils, and photographs—to facilitate emotional engagement and memory recall. The program’s themes evolved progressively from self-introduction and recalling pivotal life moments to expressing emotions, fostering resilience, and envisioning future aspirations. This preparatory description situates the subsequent qualitative findings within the specific cultural and theoretical framework of the intervention. As a pilot study, the results presented herein aim to illustrate the feasibility and cultural acceptability of the life review program by summarizing participants’ subjective experiences rather than providing generalizable conclusions.

### 3.1. Participants’ Emotional Engagement During the Life Review Sessions

During the eight-week program, participants frequently described the emotional intensity of reflecting on their past experiences. Many were visibly moved during sessions and described the life review activities as both challenging and healing.

“*When I talked about my past, I finally cried. I didn’t expect it, but it felt like something loosened inside me.*”

(Participant 3)

“*The smell of the essential oils and the music brought up memories I had not thought about in years.*”

(Participant 5)

The integration of sensory elements and structured guidance supported access to suppressed emotions.

The caring environment allowed participants to feel emotionally safe. P1 stated, “It was the first time I could speak about my regrets without being judged.” P6 added, “I usually hide my feelings, but here, I felt it was okay to be vulnerable.” These responses suggest that the program, grounded in Swanson’s “knowing” and “being with” processes, facilitated meaningful emotional expression and self-recognition.

### 3.2. Social Connection and Peer Sharing as Emergent Processes

As the group sessions progressed, participants increasingly engaged with one another, forming bonds through shared storytelling. Early hesitancy gave way to openness, as noted by Participant 2:

“*I didn’t want to talk at first, but listening to others made me feel brave enough to speak.” This mutual encouragement fostered solidarity among the group.*”

(Participant 2)

“*When others nodded while I was sharing, I felt understood. I wasn’t the only one with pain.*”

(Participant 4)

“*We laughed and cried together. It reminded me that we’re all human, no matter what we’ve been through.*”

(Participant 6)

“*Even if we came from different backgrounds, our struggles made us similar. I didn’t expect to feel connected like this.*”

(Participant 5)

The peer connections reflect the “being with” and “doing for” aspects of Swanson’s framework, as participants supported one another through empathetic listening and shared presence.

### 3.3. Reframing Life Narratives and Renewed Perspective

In the final sessions, participants demonstrated narrative shifts in how they perceived their lives. The letter-writing activity and future-focused discussions helped them reframe negative self-perceptions.

“*I always thought my life was a failure, but now I see how strong I’ve been to survive.*”

(Participant 3)

“*If I could go back and talk to my younger self, I’d say: Don’t give up—you’ll make it.*”

(Participant 2)

“*I felt like I had no value before, but after all these sessions, I realize I still have stories worth telling.*”

(Participant 6)

Others expressed renewed hope and a desire to reclaim purpose.

“*Before this program, I didn’t think the future mattered,*”

(Participant 5)

“*But now I have plans. I want to see my niece grow up. Even though I’m getting older, I don’t want to give up on myself again,*”

(Participant 1)

These expressions demonstrate the power of narrative-based reflection to cultivate meaning and reframe identity, aligning with the “maintaining belief” and “enabling” caring processes.

## 4. Discussion

This study introduced the development and pilot application of a life review program grounded in Swanson’s Theory of Caring, specifically tailored to the psychological support needs of middle-aged and older adults living with HIV in Taiwan. The program was not intended to evaluate clinical efficacy but to present a theoretically grounded and culturally sensitive intervention structure and to document participants’ responses as an initial demonstration of its applicability.

By centering the program on Swanson’s five caring processes—knowing, being with, doing for, enabling, and maintaining belief—the intervention was able to create a relational and emotionally supportive space. Participants’ responses demonstrated that the structured narrative reflection not only triggered deep emotional engagement but also enabled meaning-making and increased self-acceptance. This is consistent with prior studies affirming the potential of life review therapy to foster emotional integration and identity coherence among older adults living with HIV [27,28].

The cultural relevance of the program was also critical to its reception. Incorporating sensory elements, culturally familiar metaphors, and localized storytelling helped reduce emotional barriers and foster group cohesion. As reflected in the participants’ comments, shared experiences and mutual storytelling facilitated a sense of connection and collective healing, echoing the “being with” and “doing for” elements of Swanson’s framework. These findings align with the growing recognition that psychosocial care for aging PLWHIV must go beyond symptom reduction and instead prioritize empowerment, community, and cultural identity [29].

Notably, many participants reported a reframing of their life narratives—from stories of loss and shame to ones of survival and hope. Activities such as letter-writing and goal visualization allowed participants to reimagine their futures, reflecting Swanson’s “maintaining belief” component and affirming previous evidence on narrative reconstruction and life review therapy’s potential to improve resilience [17,30].

While these findings are promising, several limitations must be acknowledged. This pilot was conducted with only six participants, limiting generalizability. The absence of formal pre- and post-intervention psychological assessments means the program’s outcomes were documented through qualitative feedback only. However, the objective of this phase was to present the design and experiential value of the program from the user’s perspective—not to establish outcome efficacy.

This study is limited by its small sample size and lack of standardized psychological assessments, which restrict the generalizability of findings. Additionally, potential confounding factors, such as participants’ diverse life histories, varying levels of social support, and differences in health status, may have influenced their experiences and responses to the intervention. However, its strengths lie in the culturally sensitive design, integration of a caring-based theoretical framework, and focus on an underserved aging population. These aspects offer a valuable foundation for future program adaptation and evaluation. Future studies should consider adopting a mixed-methods approach to quantitatively evaluate psychosocial outcomes while preserving the depth of narrative data. Tools such as PHQ-9 and WHOQOL-HIV BREF could complement interview-based insights and enhance scientific rigor [31,32]. Moreover, this program can serve as a template for adaptation to other marginalized populations, including individuals aging with other chronic conditions or those in culturally diverse care settings. Future studies may consider incorporating standardized psychological screening tools such as the PHQ-9 or WHOQOL-HIV BREF to complement observational referral methods and enhance the rigor of participant selection.

## 5. Conclusions

This study presented a culturally sensitive life review program informed by Swanson’s Theory of Caring. The program enabled participants to reflect on their life histories, express emotions, and reconstruct personal meaning in a supportive group setting. Rather than measuring intervention efficacy, this study emphasized user experiences and implementation feasibility. The findings highlight the potential of narrative-based, caring-centered tools to enhance psychological well-being among aging PLWHIV and provide a valuable foundation for future program development and adaptation.

## Figures and Tables

**Table 1:** Life review program based on Swanson’s theory of caring.

Week	Theme	Summary of Activities	Swanson’s Theory of Caring
1	My story	1. Introduce the program’s content, themes, and expectations through a video. 2. Present the first unit’s content and sequence of activities. 3. Instruct participants to perform finger exercises with essential oils for relaxation. 4. Instruct participants to fill out the “About Me” worksheet to document their background. 5. After completion of the worksheets, facilitate participants to share their background and offer mutual encouragement. 6. Use pictures to illustrate the researcher’s life journey—both positive and negative aspects—and lead participants to reflect on whether their life has been positive or negative, and why. 7. Introduce the history of HIV infection in Taiwan and yearly infection statistics. 8. Summarize the session and preview the second unit. 9. Give each participant a small gift and take individual and group photos.	Knowing
2	The moment I found out	1. Review and discuss the content of the first unit with photos. 2. Introduce the second unit’s content and sequence of activities. 3. Instruct participants to perform finger exercises with essential oils for relaxation. 4. Play a song chosen by a participant and discuss its meaning. 5. Instruct participants to fill out the “The Moment I Found Out” worksheet by documenting how they were infected, and lead them to recall and share the moment of their HIV diagnosis and their psychological state at the time, including their experiences and mood at the moment. 6. Take a tea break together, with tea chosen by Participant A. 7. Instruct participants to fill out the “Lifeline” worksheet, sharing the happiest as well as saddest moments in life, and encourage mutual support. 8. Summarize the session and preview the third unit. 9. Give each participant a small gift and take individual and group photos.	Knowing
3	My honest feelings	1. Review and discuss the content of the second unit with photos. 2. Introduce the third unit’s content and sequence of activities. 3. Instruct participants to perform finger exercises with essential oils for relaxation. 4. Play a song chosen by Participant C and discuss its meaning. 5. Introduce the development of HIV policy in Taiwan. 6. Drink “honest feelings” tea while the researcher shares their own honest feelings. 7. Invite participants to drink “honest feelings” tea and then recall and share their feelings at the time of HIV diagnosis, and discuss how it has affected their life, including relationships and work, and talk about their current feelings. 8. Take a break together, with a favorite fruit chosen by Participant A. 9. Instruct participants to fill out the “My Strengths” worksheet, sharing personal strengths, and encourage mutual support. 10. Summarize the session and preview the fourth unit. 11. Give each participant a small gift and take individual and group photos.	Being With
4	My blessing package	1. Review and discuss the content of the third unit with photos. 2. Introduce the fourth unit’s content and sequence of activities. 3. Instruct participants to perform finger exercises with essential oils for relaxation. 4. Introduce the current state of HIV medical treatment in Taiwan. 5. Play a song of encouragement and support, discussing the meaning of the lyrics. 6. Take a break together, with the favorite juice chosen by Participant F. 7. Create a “Blessing Package,” writing down personal wishes on slips of paper and placing them inside. Encourage participants to share blessings and support for one another. 8. Summarize the session and preview the fifth unit. 9. Give each participant a small gift and take individual and group photos.	Being With
5	Write a letter to myself and someone important	1. Review and discuss the content of the fourth unit with photos. 2. Introduce the fifth unit’s content and sequence of activities. 3. Instruct participants to perform finger exercises with essential oils for relaxation. 4. Play a song chosen by Participant C and discuss its meaning. 5. Introduce Taiwan’s government and social resources related to HIV, such as “Understanding AIDS for All” for eradicating discrimination, and the mandatory HIV education in schools for teachers and administrative personnel of at least 2 h per semester. 6. Use a video to explain an HIV diagnosis and its implications in personal life. 7. Encourage participants to share their understanding of HIV and discuss the impact on their lives, as well as strategies and resources that have been helpful in adapting to a life with HIV, and their concerns and expectations for the future. Participants are required to support each other. 8. Guide participants in conceiving and writing a letter to themselves and another letter to someone important as well as sharing the letters. 9. Summarize the session and preview the sixth unit. 10. Give each participant a small gift and take individual and group photos.	Doing For
6	Send a card of love	1. Review and discuss the content of the fifth unit with photos, using an essential oil spray to fill the air with a floral scent. 2. Introduce the sixth unit’s content and sequence of activities. 3. Instruct participants to perform finger exercises with essential oils for relaxation. 4. Play a song chosen by Participant E and invite her to explain why she loves it. 5. Create an emotional support card. The researcher demonstrates how to design and write the card to convey support and encouragement, making sure every participant can feel the care and support of others. 6. Encourage participants to write words of support, gratitude, blessings, or encouragement for other members or themselves in the card. 7. Demonstrate how to design and write appealing cards and underscore the importance of letter content. Provide cards, colored pens, and sticks for card decoration. 8. Provide support and recommendations on conceiving and conveying ideas by the participants. 9. Take a break together, with a favorite tea chosen by Participant F. 10. Encourage participants (volunteers) to share the content on the cards and discuss their feelings while writing. Guide participants to share encouragement, thus enhancing interaction. 11. Hold the card delivery and blessing ceremony. Participants can opt to ask the researcher to deliver the letter or give it to someone they want to support in person. The researcher, on behalf of all the people, conveys blessings to the participants and invites everyone to give their best wishes to each other. 12. Summarize the session and preview the seventh unit. 13. Give each participant a small gift and take individual and group photos.	Doing For
7	Things I am proud of	1. Review and discuss the content of the sixth unit with photos, using an essential oil spray to fill the air with a floral scent. 2. Introduce the seventh unit’s content and sequence of activities. 3. Instruct participants to perform finger exercises with essential oils for relaxation. 4. Discuss the challenges of middle-aged and older PLWH and health management. 5. Demonstrate how to share “Things I Am Proud of” and ask participants to do so briefly. Create a positive and safe atmosphere to build trust among participants. 6. Help participants imagine future goals while focusing on “Enabling.” Ask the following questions to guide thinking: What is the thing you would most like to do for yourself or others? What changes can you control or make in the future? 7. Celebrate Participant D’s birthday with cake and singing as a special event. 8. Summarize the session and preview the eighth unit. 9. Give each participant a small gift and take individual and group photos.	Enabling
8	My wish and blessing	1. Use an essential oil spray to fill the air with a floral scent. 2. Introduce the eighth unit’s content and sequence of activities. 3. Instruct participants to perform finger exercises with essential oils for relaxation. 4. Review a video that summarizes all activities. Participants are guided to reflect on the eight-week experience and share what they have learned from each week’s activities. 5. Give each participant a commemorative booklet, encouraging them to share what they have written with each other. 6. Encourage participants to face future challenges with a positive attitude and apply what they have gained from the program to their daily lives. 7. Give each participant a small gift and take individual and group photos.	Maintaining Belief

**Table 2:** General characteristics of participants (*n* = 6).

Characteristic	Range
Age (years)	47–70
Gender	3 Male (50%), 3 Female (50%)
Duration of HIV Diagnosis (years)	10–23
Mode of HIV Transmission	5 Intravenous Drug Use (83.3%), 1 Sexual Transmission (16.7%)
Living Arrangement	6 in Long-Term Care Facilities

## Data Availability

The data presented in this study are available upon request from the corresponding author.
